# *Anaplasma* spp. in dogs and owners in north-western Morocco

**DOI:** 10.1186/s13071-017-2148-y

**Published:** 2017-04-24

**Authors:** Sarah Elhamiani Khatat, Sylvie Daminet, Malika Kachani, Christian M. Leutenegger, Luc Duchateau, Hamid El Amri, Mony Hing, Rahma Azrib, Hamid Sahibi

**Affiliations:** 1 Institut Agronomique et Vétérinaire Hassan II, Rabat, Morocco; 2 Faculty of Veterinary Medicine of Ghent University, Ghent, Merelbeke Belgium; 3 College of Veterinary Medicine, Western University of Health Sciences, Pomona, CA USA; 4 Molecular Diagnostics IDEXX Laboratories, Inc. West Sacramento, Sacramento, CA 95605 USA; 5 Laboratory of the Royal Gendarmerie, Rabat, Morocco; 6 National Reference Laboratory for Anaplasma phagocytophilum, Laboratory of Clinical Biology, Queen Astrid Military Hospital, Brussels, Belgium

**Keywords:** *Anaplasma phagocytophilum*, *Anaplasma platys*, PCR, Serology, Dogs, Humans, *Rhipicephalus sanguineus*, Morocco

## Abstract

**Background:**

*Anaplasma phagocytophilum* is an emerging tick-borne zoonotic pathogen of increased interest worldwide which has been detected in northern Africa. *Anaplasma platys* is also present in this region and could possibly have a zoonotic potential. However, only one recent article reports on the human esposure to *A. phagocytophilum* in Morocco and no data are available on canine exposure to both bacteria. Therefore, we conducted a cross-sectional epidemiological study aiming to assess both canine and human exposure to *Anaplasma* spp. in Morocco. A total of 425 dogs (95 urban, 160 rural and 175 working dogs) and 11 dog owners were sampled from four cities of Morocco. Canine blood samples were screened for *Anaplasma* spp. antibodies by an enzyme-linked immunosorbent assay (ELISA) and for *A. phagocytophilum* and *A. platys* DNA by a real-time polymerase chain reaction (RT-PCR) targeting the *msp*2 gene. Human sera were tested for specific *A. phagocytophilum* immunoglobulin G (IgG) using a commercial immunofluorescence assay (IFA) kit.

**Results:**

*Anaplasma* spp. antibodies and *A. platys* DNA were detected in 21.9 and 7.5% of the dogs, respectively. *Anaplasma phagocytophilum* DNA was not amplified. *Anaplasma platys* DNA was significantly more frequently amplified for working dogs. No statistically significant differences in the prevalence of *Anaplasma* spp. antibodies or *A. platys* DNA detection were observed between sexes, age classes or in relation to exposure to ticks. A total of 348 *Rhipicephalus sanguineus* (*sensu lato*) ticks were removed from 35 urban and working dogs. The majority of dog owners (7/10) were seroreactive to *A. phagoyctophilum* IgG (one sample was excluded because of hemolysis).

**Conclusions:**

This study demonstrates the occurrence of *Anaplasma* spp. exposure and *A. platys* infection in dogs, and *A. phagocytophilum* exposure in humans in Morocco.

## Background

Ticks are considered to transmit the widest number of pathogens when compared to other arthropod vectors, and several of these pathogens are of veterinary and medical importance [1]. Some tick-borne pathogens (TBPs) are considered to be emerging because of several factors that play a crucial role in ticks multiplication and expansion, increasing the likelihood of ticks feeding on humans and animal and transmitting pathogens [2]. Among these emerging TBPs of zoonotic relevance, *Anaplasma phagocytophilum* (formerly *Ehrlichia equi*, *Ehrlichia phagocytophila*, and the human granulocytic ehrlichiosis agent) is an obligate intracellular gram-negative bacterium belonging to the family of *Anaplasmataceae* [3]. This bacterium causes a widespread disease called granulocytic anaplasmosis and is commonly transmitted by *Ixodes* tick species [4]. In the past decades, both human and animal exposure to *A. phagocytophilum* has continuously increased in the USA, Europe and some Asian countries [4–8]. The clinical presentation of human granulocytic anaplasmosis is a non-specific flu-like disease potentially fatal with severe complications, high hospitalization rates and difficult diagnosis [7–9]. Dogs are mostly recognized as incidental hosts and their role as potential reservoir hosts for *A. phagocytophilum* infection is still controversial [10]. However, some authors suggested that dogs may be considered as potential reservoir hosts for *A. phagocytophilum* in some regions, especially in urban environments [11–14], or at least as effective sentinels to assess the risk for human infection [15].

*Anaplasma platys* is another species of *Anaplasma* known to infect dogs, which are considered the main reservoir hosts. This bacterium is most likely transmitted by *Rhipicephalus sanguineus* (*s.l*.) ticks and is responsible for infectious canine cyclic thrombocytopenia [16]. *Anaplasma platys* is not considered as zoonotic although infection of other domestic animals [17–22] and humans [23–27] have been reported. Both *A. platys* and *A. phagocytophilum* infections remain usually asymptomatic or subclinical in dogs. When present, clinical signs are unspecific and include fever, lethargy, anorexia, lymphadenopathy, lameness, thrombocytopenia and anemia [15, 16].

In Morocco, both *Ixodes* spp. and *R. sanguineus* (*s.l*.) ticks are present [28–30]. In addition, *A. phagocytophilum* and *A. platys* were reported in domestic animals and ticks in North Africa [31–36]. However, only one recent report described human exposure to *A. phagocytophilum* in Morroco [37] and no data are available on the canine exposure to both *A. phagocytophilum* and *A. platys*. Therefore, the aim of this study was to assess the occurrence of *Anaplasma* spp. infection and/or exposure in different groups of dogs and dog owners in Morocco.

## Methods

### Dogs

Between December 2013 and May 2015, 425 dogs were sampled from four Moroccan cities and divided in 3 groups. The first group (Group I) included 95 client-owned dogs sampled in the Veterinary Teaching Hospital (VTH) of the Institut Agronomique et Vétérinaire Hassan II, Rabat (34°01'31"N, 06°50'10"W). These dogs were clustered in two subgroups: Group Ia included 63 dogs without clinical signs compatible with tick-borne diseases (TBDs) and brought to the VTH for vaccination, surgery or post-surgical follow up, dermatology, cardiology or orthopedic consultations, and Group Ib included 32 dogs with clinical signs compatible with TBDs (fever, inapetence or anorexia, lethargy and lameness without orthopedic origin). For each dog of the first group, an epidemiological questionnaire was completed describing the date of sample collection, age, sex, breed, outdoor activities, ectoparasite prophylaxis, exposure to ticks, travel history outside Morocco during the previous year, vaccination status, presenting complains and physical examination. The second group (Group II) was composed of 160 client-owned dogs from the rural region of Sidi Kacem (34°13'00"N, 5°42'00"W). These dogs behave like stray or roaming dogs because of their outdoor living, close contact with other domestic of feral animals, and low health and or wellness care (absence or irregular vaccination and/or, parasite prevention). Information available on this group included age, sex and breed. The third group (Group III) contained 170 military and gendarmerie working dogs sampled in the first kennel of the Royal Army Forces of Benslimane (33°36'44"N, 7°07'16"W) and the kennel of the Royal Gendarmerie of Temara (33°55'36"N, 6°54'44"W), respectively. Data available on these dogs were age, sex and breed. Groups II and III included apparently healthy dogs considered at high risk for acquiring TBPs because of their regular outdoor activities or permanent outdoor living conditions and irregular ectoparasites prevention. All owners gave their consent for enrollment of their dogs.

For each dog, 8 ml of non-anticoagulated blood were collected from the cephalic vein. Blood was centrifuged at 3,500× *rpm* for 10 min and serum was separated, aliquoted and frozen at -32 °C. In addition, 2 ml of whole blood collected on ethylenediaminetetra-acetic acid (EDTA) anticoagulant tubes were sampled and frozen at -32 °C. The frozen sera and whole blood samples were sent to the IDEXX Laboratories (Sacramento, California, USA) to be tested for for anti-*Anaplasma* spp. antibodies and for *A. phagocytophilum* and *A. platys* using PCR.

### Ticks

A total of 348 ticks were removed manually from the dogs included in this study, identified (species, stage, sex) [38] and conserved in 70% ethanol at 4 °C until shipment to the IDEXX Laboratories (Sacramento, California, USA).

### Owners

All dog owners of the dogs included in Group I were contacted by phone to be sampled for *A. phagocytophilum* antibodies testing. Only eleven accepted to be enrolled in this study and signed an informed consent forms. An epidemiological report was completed for each owner. Age, city of residence, occupational activity, travels outside Morocco during the previous year, outdoor activities, tick exposure and potential contact with dogs and other domestic animals (cats, horses and ruminants) were recorded.

For each patient, 5 ml of non-anticoagulated blood were collected from the elbow groove vein. Blood samples were centrifuged at 3,500× *rpm* for 10 min and serum was separated, aliquoted and stored at -32 °C until shipment to the National Reference Laboratory for *A. phagocytophilum* in Queen Astrid Hospital (Brussels, Belgium).

### Laboratory procedures

#### Serological analysis of canine sera (ELISA)

The *Anaplasma* spp. antibody ELISA utilizes orthogonal assay protocols to screen and subsequently confirm the presence of *Anaplasma* antibodies in a serum or plasma sample. The protocols employ microwells coated with *Anaplasma* p44 peptide and *Anaplasma* peptide conjugated to Horseradish peroxydase (HRPO) [39]. Briefly, 50 μl of sample was added to a microtiter plate well, followed by 50 μl of conjugate. The plate was incubated for 30 min at room temperature. Wells were washed 5 times with a PBS Tween wash solution, followed by adding 100 μl of TMB substrate and a 15-min incubation step at room temperature. The assay is stopped by adding a stop solution and read at 650 nm using a plate reader spectrophotometer. Positive and negative controls were run in parallel on each plate.

#### DNA extraction and real-time PCR assays on dogs

EDTA blood samples were used to extract total nucleic acid following a protocol adapted from Boom et al. [40]. Briefly, 180 μl whole blood were resuspended in a lysis solution and incubated for 10 min. Lysates were extracted using Whatman binding plates (Thermo Fisher Scientific, Whatham, Massachusetts, USA) on a Corbett X-Tractor platform (Qiagen, Valencia, CA, USA). Nucleic acids were eluted into 150 μl of PCR-grade nuclease-free water (Thermo Fisher Scientific, Whatham, Massachusetts, USA) and 5 μl amplified in subsequent real-time PCR reactions. Analysis was performed on a Roche LightCycler 480 (Roche Applied Science, Indianapolis, USA) and raw data analyzed using the second derivative maximum method with the ‘high sensitivity’ setting to generate crossing points (CP values).

Whole blood samples for PCR testing were available only for 362 dogs including 59 from Group Ia, 32 from Group Ib, 104 from Group II and 167 from Group III. *Anaplasma* spp. real-time PCR assays were used from a commercial source (IDEXX Laboratories, Inc., Westbrook, Maine, USA; test code 2824 RealPCR^TM^ test). Real-time PCR tests were designed using a commercially available software (PrimerExpress 3.0) according to the published guidelines [41]. The test was adapted from previous publications [42, 43] and consisted of a mixture of two strain specific tests including *A. phagocytophilum* (*msp*2 gene, GenBank accession no. DQ519570) and *A. platys* (AY848753). PCR tests positive for *Anaplasma* spp. were then screened at the species level using the individual strain specific real-time PCR tests. The internal sample control real-time PCR test was designed using 18S rRNA (DQ287955). All assays were designed and validated according to industry standards (Thermo Fisher Scientific, Whatham, Massachusetts, USA; User Bulletin #3).

Real-time PCR was run with 6 quality controls including (i) PCR positive controls (quantitatively); (ii) PCR negative controls; (iii) negative extraction controls; (iv) DNA pre-analytical quality control targeting canine 18S rRNA gene complex; (v) environmental contamination monitoring control; and (vi) spike-in internal positive control. These controls assessed the functionality of the PCR test protocols for the (i), absence of contamination in the reagents (ii) and laboratory (v), absence of cross-contamination during the extraction process (iii), quality and integrity of the DNA as a measure of sample quality (iv), reverse transcription protocol (v and vi) and absence of PCR inhibitory substances as a carryover from the sample matrix (vi).

Real-time PCR tests were validated analytically and clinically. For the analytical validation, each assay had to pass 6 validation criteria including amplification efficiency, linearity, reproducibility intra-run, reproducibility inter-run, r-square value and signal to noise ratio of the fluorescent signal. Clinical samples were used to repeat standard curves and to confirm PCR positive results by sequencing with outside flanking primers. A total of 4,125 clinical samples were used during the clinical validation of this panel and test results were compared to either alternative PCR test systems or immunofluorescence assay (IFA) methods.

#### Serological analysis of human sera (IFA)

**Fig. 1:**
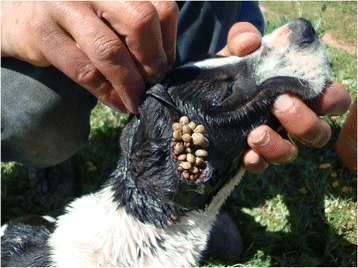
*Rhipicephalus sanguineus* engorged ticks attached to the ear of a dog from Group I

Human sera were screened for *A. phagocytophilum* immunoglobulin G (IgG) antibodies by a semi-quantitative indirect IFA using a commercial kit (Focus Diagnostics, Cypress, California, USA) containing HL60 cells infected with a human isolate of *A. phagocytophilum* HGE-1 according to the manufacturer’s instructions. Briefly, 5 μl of serum were diluted in 315 μl of phosphate-buffer saline (PBS) (0.01 M, pH = 7.2 ± 0.1). The positive IgG control was also diluted in PBS to obtain five dilutions 1:2, 1:4, 1:8, 1:16 and 1:32. Then, 25 μl of diluted sera were added in the slides wells (one well per sample). The first line of the first slide contained the negative IgG control and the five dilutions of the positive IgG control. The slides were incubated in humid chambers between 35.0 and 36.5 °C for 30 min. After the incubation period, the slides were washed with PBS solution followed by distilled water to eliminated non conjugated serum antibodies. In the second step, 25 μl of conjugate containing human IgG combined with fluorescein were added in each well. The slides were incubated again then washed in the same formerly described conditions. Finally, the slides were dried, coverslipped using mounting medium and observed with ultraviolet light microscopy (×400). The titer was defined as the reciprocal of the highest dilutions of serum with the homogeneously stained cytoplasmic morulae (Fig. 1). A serum titer of ≥ 1:64 was considered as positive for *A. phagocytophilum* IgG according to the manufacturer’s instructions. Samples that were positive at the first dilution of 1:64 under ultraviolet light microscopy (×400) were then further diluted to 1:128 and those remaining positive at the second dilution were then tittered at 1:256 and 1:512.

### Statistical analysis

Statistical analysis was performed using SAS version 6.4 (SAS Institute Inc., Car, NC, USA). The exact logistic regression model was fitted to compare seroreactivity and PCR positive rates between the different groups, age classes, sex and in relation to the presence of ticks. First, global hypothesis tests were performed, comparing all dog groups, based on the likelihood ratio test (LRT). With an overall significant test, groups were compared pairwise using Bonferroni’s multiple comparisons technique at a global significance level of 5%. Significant pairwise comparisons were summarized in terms of the odds ratio (OR) with a 95% confidence interval (95% CI). Other risk factors (sex, tick exposure, age groups) were analysed in the same way.

## Results

### Serological and molecular screening of dogs

Off the 425 dogs, breed, sex and age were available for 299 (70.3%), 398 (93.6%) and 402 (94.6%) dogs, respectively. Dogs belonged to 23 different breeds with German and Belgian Shepherds (*n* = 122), Retrievers (*n* = 58), Saluki (*n* = 36), Cocker and English Spaniel (*n* = 27), mixed breeds (*n* = 19) and Pointers dogs (*n* = 10) the most frequently found during sampling. Other breeds included Poodles (*n* = 4), Rottweilers (*n* = 3), Pekingese (*n* = 3), Aidi (*n* = 2), Border Collie (*n* = 2), Pitbull (*n* = 2), Setters dogs (*n* = 2) and one dog for Drahthar, Saint Hubert, German Mastiff, Argentin dogo, Dalmatian, Akita Inu, Husky, Havanese and Chihuahua. The age of dogs ranged from 3 months to 14 years-old (mean age 3.2 years-old) and males (*n* = 257) were more frequently sampled than females (*n* = 141). Previous ticks bites were available for 226 dogs (53.2%) from Group I (*n* = 40) and Group III (*n* = 18).

Table 1 summarises the results of *Anaplasma* spp. antibodies and *A. platys* DNA detection in the three groups of dogs. There were significant differences between dog groups (*χ* ^2^ = 10.28, *df* = 3, *P* = 0.016). Group Ia differed significantly from Group II (OR = 0.32, 95% CI: 0.14–0.75, *P* = 0.009). None of the 362 dogs screened for *A. phagocytophilum* DNA by PCR was found positive whereas 7.5% (95% CI: 0.05–0.11) of them were positive to *A. platys* (Table 1). There were globally significant differences between dog groups (*χ* ^2^ = 9.44, *df* = 3, *P* = 0.024). The highest prevalence of *A. platys* DNA detection was found in Group III but none of the pairwise comparisons was significant (Table 1). Table 2 summarizes the prevalence of positivity rates to *Anaplasma* spp. antibodies and *A. platys* DNA detection according to sex, age and exposure to ticks. No statistically significant differences were found in seropositivity rates for the sex (*χ* ^2^ = 2.161, *df* = 1*, P* = 0.142), the age groups (*χ* ^2^ = 1.75, *df* = 2, *P* = 0.416) and exposure to ticks *(χ* ^2^ = 0.83, *df* = 1, *P* = 0.363). Similarly, no statistically significant differences were found in positivity rates to *A. platys* DNA detection for sex (*χ* ^2^ = 2.88, *df* = 1*, P* = 0.090), the exposure to ticks and age groups (*χ* ^*2*^ = 5.05, *df* = 2, *P* = 0.080).

**Table 1:** Number and prevalence (%) of positive and negative dogs to *Anaplasma* spp. antibodies (by ELISA) and *A. platys* DNA detection (by PCR), and positive to both methods in the different groups

Group^a^	*Anaplasma* spp. antibodies (%) (*n* = 425)		*A. platys* (%) (*n* = 362)			*Anaplasma* spp. and *A. platys* (%) (*n* = 362)
	Positive	Negative	Positive	Negative	Not available	
Group I (*n* = 95)	11 (2.6)	84 (19.8)	3 (0.8)	88 (24.3)	4	1 (0.3)
Group Ia (*n* = 63)	7 (1.6)	56 (13.2)	2 (0.5)	57 (15.7)	4	0 (0.0)
Group Ib (*n* = 32)	4 (0.9)	28 (6.6)	1 (0.3)	31 (8.6)	0	1 (0.3)
Group II (*n* = 160)	45 (10.6)	115 (27.1)	4 (1.1)	100 (27.6)	56	1 (0.3)
Group III (*n* = 170)	37 (8.7)	133 (31.3)	20 (5.5)	147 (40.7)	3	9 (2.3)
Total (*n* = 425)	93 (21.9)	332 (78.1)	27 (7.5)	335 (92.5)	63	11 (3.0)

^a^Group I: urban client-owned dogs sample in the VTH; Group Ia: urban client-owned dogs sample in the VTH without clinical signs compatible with a TBD; Group Ib: urban client-owned dogs sample in the VTH with clinical signs compatible with a TBD; Group II: rural client-owned dogs; Group III: military and gendarmerie working dogs

**Table 2:** Number and prevalence of (%) positive and negative dogs to *Anaplasma* spp. antibodies (by ELISA) and *A. platys* DNA detection (by PCR) according to the sex, the age and the exposure to ticks

Variable		*Anaplasma* spp. antibodies (%) (*n* = 425)			*A. platys* DNA (%) (*n* = 362)		
		Positive	Negative	Not available	Positive	Negative	Not available
Sex	Male	59 (13.9)	198 (46.6)	–	20 (5.5)	187 (51.7)	50
	Female	23 (5.4)	118 (27.8)	–	5 (1.4)	123 (34.0)	13
Age (yrs)	< 1	9 (2.1)	52 (12.2)	–	3 (0.8)	52 (14.4)	6
	1–5	56 (13.2)	194 (45.6)	–	21 (5.8)	183 (50.6)	46
	≥ 6	13 (3.0)	61 (14.3)	–	2 (0.5)	62 (17.1)	10
Ticks exposure		40 (9.4)	46 (10.8)	9	40 (11.0)	46 (12.7)	9

A total of 348 ticks were removed from 35 dogs and all belonged to *R. sanguineus* (*s.l*.). Two ticks were nymphs, 284 adult females and 63 adult males. The number of ticks removed from one dog ranged from 1 to 54 (mean number 9.9) (Fig. 1). Among the 35 infested dogs, 15 belonged to Group I, 2 to Group II and 18 to Group III. The number of dogs infested by ticks and positive to *Anaplasma* spp. antibodies only, to *A. platys* DNA only or to both tests were eight, three and one, respectively. The only dog infested by ticks and positive for both tests was from Group II.

### Serological screening of owners

Among the eleven dog owners sampled, three were women and eight were men. Ages ranged from 23 to 66 years, with an average of 51 years. Most lived in Rabat (9/11) and two in surrounding cities (Salé and Arjat). Seven mentioned having leisure outdoor activities in forest or rural areas and one farmer lived in a rural area (Arjat). Five owners reported to have contact with other domestic animals including cats, horses and ruminants. Five owners had additional dogs. Only one owner reported previous exposure to ticks and two traveled to foreign countries during the year.

**Fig. 2:**
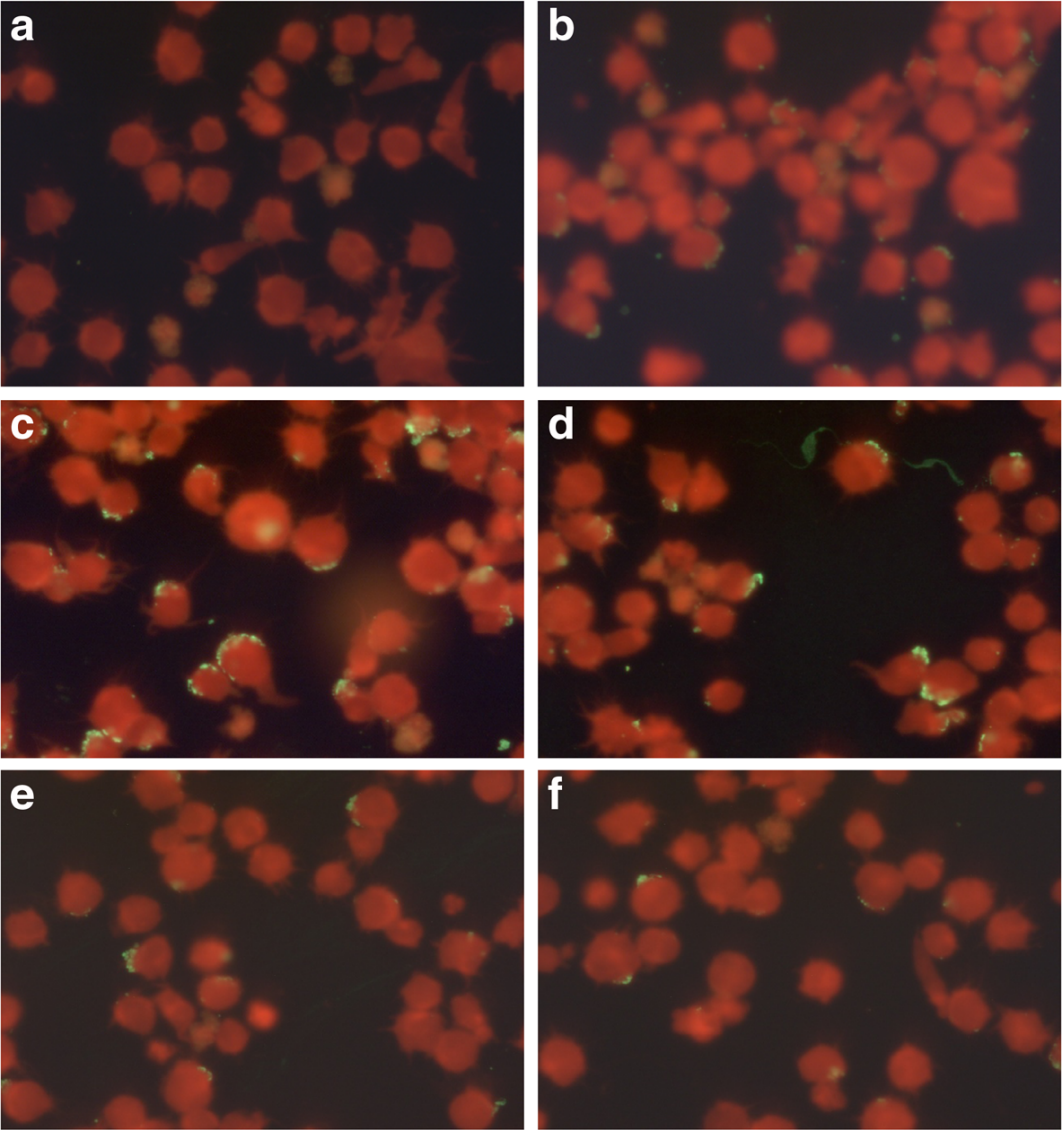
Photographs of ultraviolet light microscopy (×400) of *A. phagocytophilum* IgG using IFA and showing a negative control (**a**), a positive control (**b**) and for positive dilutions i.e. 1:64 (**c**), 1:128 (**d**); 1:256 (**e**) and 1:516 (**f**). The positivity is set on the observation of green morulae surrounding the cell’s cytoplasmic membrane

One sample was excluded due to hemolysis that could interfere with the results according to the manufacturer’s instructions. Seven out of the ten remaining sera were positive to *A. phagocytophilum* IgG at the first dilution (1:64) (Fig. 2). Among the seropositive owners, three were women and four were men. Four reported regular outdoor activities in the forests of Rabat or the vicinity (Maamora forest, Khémisset, Bouznika and Benslimane). Four owners mentioned to have contact with domestic animals other than dogs. None of the seropositive owners had a travel history outside Morocco during the previous year and two mentioned to be regular blood donors. When further diluted, six, two and one samples remained positive at 1:128, 1:256 and 1:512, respectively (Fig. 2). The only sample that remained positive at 1:512 was from a farmer.

## Discussion

To our knowledge, the results of this cross-sectional study demonstrated for the first time in Morocco a prevalence of 21.9 and 7.5% of *Anaplasma* spp. antibodies and *A. platys* DNA detection in dogs, respectively. It also showed that 7 among 10 dog owners were seroreactive to *A. phagocytophilum* IgG. Currently the two most important *Anaplasma* species known to infect dogs and humans are *A. platys* and *A. phagocytophilum* [27]. Infection by both species have already been detected in dogs and ticks in North Africa [28, 31, 33–36]. Our study detected *A. platys* infection in dogs with a prevalence similar to what has been published in Algeria (5.4%) [36]. Although not statistically significant, working dogs tested more frequently positive to *A. platys* DNA than rural dogs. Therefore, although considered as a major risk factor for acquiring tick-borne infections [44–46], outdoor access alone cannot explain the high prevalence in working dogs. Similarly, a study on Senegalese gendarmerie and private kennel living dogs showed a high prevalence of *E. canis* infection [47], another *R. sanguineus* (*s.l*.)-transmitted pathogen, probably because this tick species can complete its entire life-cycle either indoor (in houses, kennels and veterinary hospitals where it readily colonizes the infrastructure) or in outdoor environments (peri-urban and rural) [25, 47, 48]. Other factors explaining the higher prevalence in working dogs in our study can be the absence of efficient ectoparasite control programs in this group or the access to areas with higher burdens of *A. platys*.

Our study detected both *Anaplasma* spp. antibodies and *A. platys* DNA in dogs but failed to identify *A. phagocytophilum* DNA. This discrepancy has also been reported in other African, European and American studies [31, 49–51]. Cross-reactivity between *Anaplasma* spp. pathogens, especially between *A. phagocytophilum* and *A. platys,* has been reported to occur. Therefore, in regions were both pathogens could co-exist, seropositivity may not enable the distinction at the species level [16]. In areas where *Ixodes* spp. ticks, are less prevalent or absent, a positive *Anaplasma* spp. serology could be the result of *A. platys* exposure [52]. Consequently, the fact that we detected exclusively *R. sanguineus* (*s.l*.) ticks infesting dogs can be supportive of the potential predominance of *A. platys* in Morocco. However, *Ixodes* spp. ticks are also present in this country [28–30] and could have infected these dogs previously. On the other hand, infection with *A. phagocytophilum* in *Rhipicephalus* spp. has also been reported especially in the Mediterranean countries, and these ticks have been suggested as potential competent vectors of this bacterium in this part of the world [33, 53–56]. In a study from Jordan, a high prevalence of *A. phagocytophilum* infection (39.5%) was found in dogs and the most abundant tick species removed was *R. sanguineus* (*s.l*.) (95.1%) followed by two *Haemaphysalis* species, whereas no *I. ricinus* was collected from these dogs. The authors suggested that the ticks found in their study could be a possible competent vector of the pathogens detected including *A. phagocytophilum* [57]. Further studies are necessary to evaluate the ability of *Rhipicephalus* ticks in transmitting *A. phagocytophilum*.

In regions where both *A. platys* and *A. phagocytophilum* are present, a PCR-based assay is required to determine which of the two agents is responsible for positive serological test [16]. Nevertheless, false-negative results are reported to occur with PCR, mainly due to low template concentrations [27, 58], the short duration of *A. phagocytophilum* bacteremia in dogs and the variations in the levels of circulating bacteria [15, 58]. In addition, selective amplification of the predominant organism can occur in patients coinfected with genetically similar organisms [27, 59] such as *A. phagocytophilum* and *A. platys,* which could be the case in our study. As DNA-based diagnostic tool enables the early detection of the infection by *A. phagocytophilum*, the bacteriemia is of short duration and is usually present transiently during the acute phase of the infection [15, 60, 61], negative PCR results might be more difficult to interpret in healthy dogs. Therefore, negative PCR results only indicate that the respective nucleic acid sequence was not detected in the sample evaluated under the assay conditions used and should not be interpreted as evidence of absence of infection [58]. In addition, other factors could explain the negative results in our study mainly the likely degradation of the DNA due to the transport conditions from Morocco to the USA and the selected region of sampling. Indeed, our dogs were sampled exclusively from the western part of Morocco but previous studies detected *I. ricinus* ticks in the eastern regions [28, 29]. In addition, *Borrelia burgdorferi* (*s.l*.), that is transmitted by *Ixodes* spp. ticks, was reported in dogs in Algeria [31], a neighbour country of Morocco, and ticks in north-eastern Morocco [30], suggesting that these ticks could be more prevalent in eastern regions.

Consistently with our previous report that detected high prevalence rates of *A. phagocytophilum* exposure in humans in northwestern regions of Morocco [37], the majority of dog owners sampled were found positive to *A. phagocytophilum* IgG. In our previous study, the contact with dogs or other domestic animals was not a risk factor for the seropositivity [37], suggesting that other factors such as outdoor activities might be incriminated. Indeed, outdoor activities especially related to forests, meadow habitats and grasslands are considered as a major risk factor for acquiring a tick-borne infection due to the increase risk of contact with infected ticks [62]. Another study has found no significant difference in the seroprevalence of *A. phagocytophilum* among owners of seropositive pets and owners without pets, suggesting that dog ownership may not be a risk factor [63].

*Anaplasma platys* was known to infect dogs exclusively, and they are are recognized as the main reservoir hosts. However, recent reports described the infection in domestic ruminants, cats and even in humans [17–27]. In addition, human infestation with *R. sanguineus* (*s.l*.) has also been reported [47, 57, 59], suggesting that *A. platys* could be transmitted to humans through the bite of this tick species. Moreover, all human cases infected with *A. platys* had regular contact with dogs and/or reported infestation of their dogs with *R. sanguineus* (*s.l*.) [25–27]. In addition, in two human cases, the *A. platys* sequence was identical to the sequence found in their dog [27]. This is in contrast to our current and previous study that both failed to detect a relationship between contact with dogs and human seropositivity to *A. phagocytophilum* possibly suggesting that humans in Morocco could be more likely to exposed to this bacterium than to *A. platys*. All previously reported cases of human *A. platys* infection were diagnosed by DNA detection or microscopic identification of morulae within platelets [25–27] and hence, the occurrence of immunological response to this bacterium is unknown. Moreover, to the authors’ knowledge, the possible occurrence of cross-reaction between *A. platys* and *A. phagocytophilum* antibodies has not been evaluated in humans. The IFA based on HL60-cells infected with a human isolate of *A. phagocytophilum*, such as the one used in our study, are considered to be both sensitive [64] and highly specific for the investigation of seroreactivity to this bacterium [9] with a specificity of 100%, according to the manufacturer.

*Rhipicephalus sanguineus* (*s.l*.) is the most common tick in the Mediterranean region [57]. It is known to transmit several pathogens including *Rickettsia conorii, Babesia canis, Hepatozoon canis* and *E. canis* and probably *Bartonella* spp., *Mycoplasma haemocanis* and *A. platys* [46]. This tick has the particularity to be active during almost all the year and to achieve two or more generations per year. Warmer temperature may contribute to an increased tick abundance by a more rapid development. Although *R. sanguineus* (*s.l*.) ticks usually feed on dogs, they can feed on a wide variety of animal species including humans [48, 65]. Therefore, due to its high degree of adaptability, *R. sanguineus* (*s.l*.) represents a major threat not only to dogs, but also to humans. Furthermore, the report of *E. canis* and *A. platys* human infections [23–27, 66, 67] emphasizes the importance of *R. sanguineus* (*s.l*.) and the zoonotic potential of these two infections, and further investigation should be carried out to assess the public health implication [48].

The major limitations of this study are the restricted area of sampling, the absence of PCR performed on the ticks sampled from dogs, and the small number of owners and dogs with clinical signs compatible with a TBD. Unfortunately, DNA from the ticks collected was too degraded to perform PCR analysis, most probably due to the shipping conditions from Morocco to the USA.

## Conclusions

This study demonstrates the *Anaplasma* spp. exposure in humans and dogs in Morocco. To our knowledge, it is also the first report on the occurrence of *A. platys* infection in dogs. Our results showed that working dogs living in kennels are at an increased risk for acquiring this infection. These findings highlight the importance of regular preventive measures against arthropod vectors especially in dogs living in kennels and dogs that have access to outdoor environments. This study also suggets that human exposure to *A. phagocytophilum* is likely to be frequent and emphazises the need for large-scale serological and clinical surveys to better estimate the prevalence of this bacterium and to determine its ability in causing disease in Morocco. Since the human infection by *A. platys* has been reported, Moroccan dogs are frequently infected with this bacterium and dogs are the main reservoir hosts, it is important to evaluate if this bacterium can cause human disease in Morocco and if the infection is associated with an immunological response. This study should serve as an indicator to Moroccan physicians and veterinarians that *A. phagocytophilum* and *A. platys* exposure and infection are not rare, and it will help raise awareness on the potential occurrence of TBDs more generally in this country. Since we reported results in a limited area of the country and on a very limited number of humans, larger and more represeantative surveys are recommended.

## Abbreviations

EDTA: Ethylenediaminetetra-acetic acid
ELISA: Enzyme-linked immunosorbent assay
IFA: Immunofluorescence assay
IgG: Immunoglobulin G
RT-PCR: Real-time polymerase chain reaction
TBD: Tick-borne disease
TBPs: Tick-borne pathogens
